# Severe Nocardia pneumonia in an immunocompromised patient with alpha‐1 antitrypsin deficiency

**DOI:** 10.1002/rcr2.670

**Published:** 2020-09-24

**Authors:** Daryl E. C. Y. Chan, Dima Hamed, Daniel Lennon, Peter Wark

**Affiliations:** ^1^ Department of Respiratory and Sleep Medicine John Hunter Hospital Newcastle NSW Australia; ^2^ NSW Health Pathology – Hunter Newcastle NSW Australia; ^3^ Centre for Healthy Lungs University of Newcastle Newcastle NSW Australia

**Keywords:** Alpha‐1 antitrypsin deficiency, corticosteroids, emphysema, immunocompromised, Nocardia

## Abstract

Pulmonary nocardiosis is an uncommon pulmonary infection that is more likely found in immunocompromised patients with underlying chronic lung disease. The presentation of pulmonary nocardiosis is widely variable and shares features with other types of pulmonary infections. Nocardia is also not as easily isolated on standard culture mediums and hence more difficult to identify. We describe the case of a patient with a severe necrotising pneumonia who was chronically immunosuppressed with steroids and has alpha‐1 antitrypsin deficiency.

## Graphical abstract

Pulmonary nocardiosis is an uncommon pulmonary infection that is more likely found in immunocompromised patients with underlying chronic lung disease. The presentation of pulmonary nocardiosis is widely variable and shares features with other types of pulmonary infections. Nocardia is also not as easily isolated on standard culture mediums and hence more difficult to identify. We describe the case of a patient with a severe necrotizing pneumonia who was chronically immunosuppressed with steroids and has alpha‐1 antitrypsin deficiency.

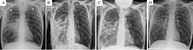

## Introduction

Nocardia species are Gram‐positive rods that are normally found in soil and water. They commonly cause infections in immunocompromised people but can also occur in immunocompetent patients. Nocardia can cause either localized disease (mainly pulmonary or cutaneous) or disseminated disease. It can spread to almost any organ but most commonly disseminates to the central nervous system. As pulmonary nocardiosis presents similarly to other pulmonary infections both clinically and radiologically [1] and given its longer incubation time, Nocardia may not be as readily identified as the causative organism in pulmonary disease [2].

We describe the case of a patient who was chronically immunosuppressed on high‐dose corticosteroids and has alpha‐1 antitrypsin deficiency who had a severe Nocardia pneumonia.

## Case Report

A 36‐year‐old man was referred from a peripheral hospital with a three‐week history of progressive dyspnoea, fatigue, productive cough, and right‐sided pleuritic chest pain. He had a background history of chronic obstructive pulmonary disease secondary to alpha‐1 antitrypsin deficiency (PiZZ genotype), which was discovered the previous year when he had presented with a spontaneous pneumothorax and was found to have significant basal panacinar emphysema given his age. Despite the diagnosis, he never followed up with a respiratory physician and continued smoking. He had also managed to obtain access to a large amount of corticosteroids and had been taking 50 mg of prednisolone daily (1 mg/kg in the context of the patient weighing 48 kg) whilst continuing to smoke for a total of six months prior to his presentation.

**Figure 1:**
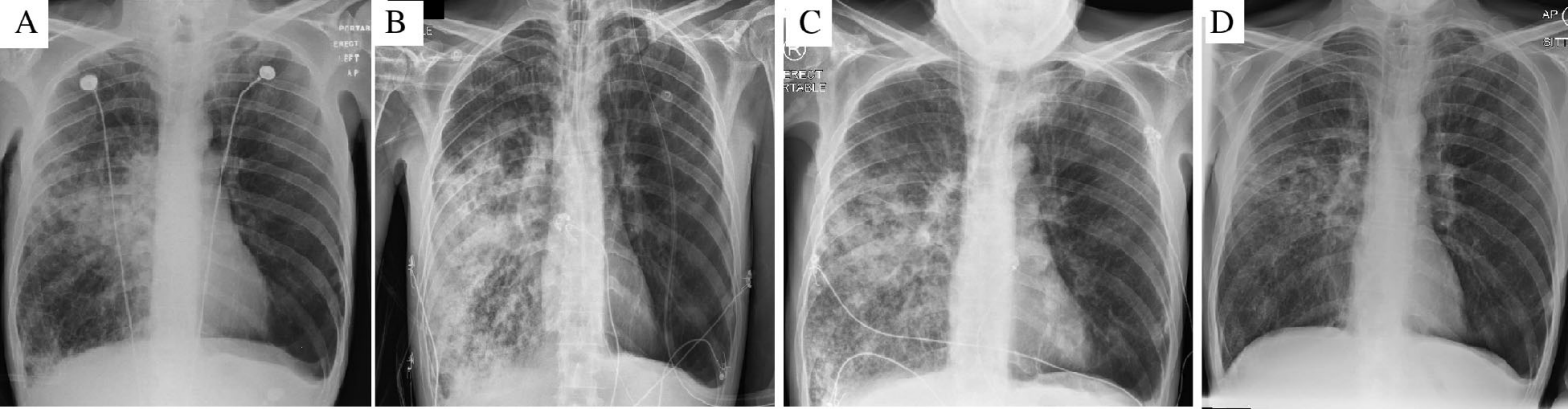
Serial chest X‐rays showing progressive worsening and then resolution of consolidation in the right perihilar region and mid to lower zone. Also of note is the significant amount of basal gas trapping more pronounced on the left side in keeping with the patient's diagnosis of alpha‐1 antitrypsin deficiency‐associated emphysema. (A) Day 1 of admission, (B) day 4 of admission, (C) day 9 of admission, and (d) day 17 of admission (prior to discharge).

His initial investigations revealed a white blood cell count of 18.5 × 10^9^/L with neutrophilia (17.8 × 10^9^/L) and lymphopaenia (0.5 × 10^9^/L). C‐reactive protein (CRP) was markedly elevated at 516 mg/L (reference range < 5 mg/L). His chest X‐ray showed hyperinflated lungs with patchy consolidation in the right middle zone and perihilar region (Fig. 1). Initially, he received empirical antibiotics for a severe community‐acquired pneumonia (ceftriaxone and azithromycin) and stress‐dose steroids. Preliminary sputum samples were sent for microscopy and culture.

**Figure 2:**
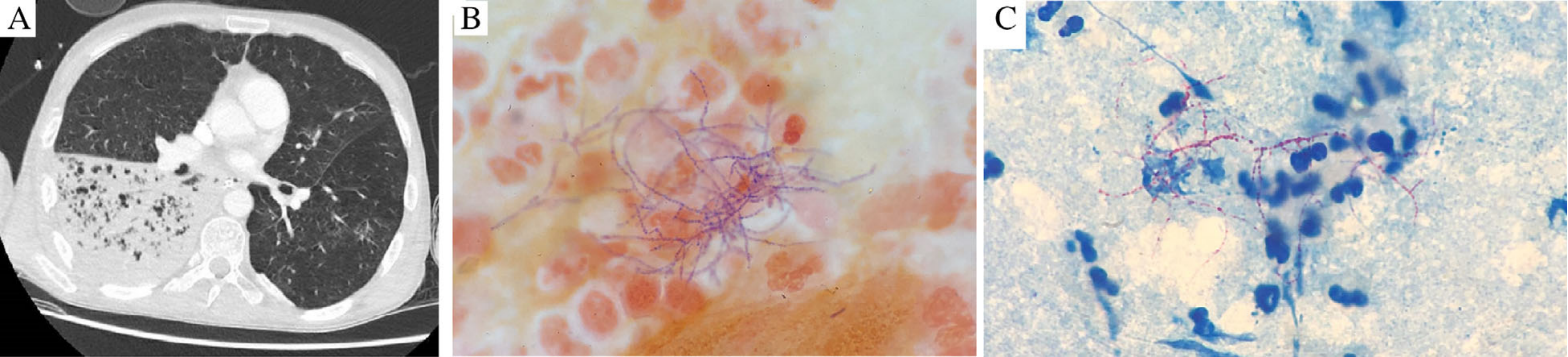
(A) Computed tomography (CT) of the thorax showed a lobar pneumonia involving the right lower lobe. Numerous small locules of gas can be seen within the consolidated lung. CTs of the brain, abdomen, and pelvis did not reveal any disseminated Nocardia. (B) Gram‐positive branching rods on sputum Gram stain identified as Nocardia. (C) Acid‐fast branching rods on modified Ziehl–Neelsen stain identified as Nocardia.

Despite therapy, the patient continued to deteriorate over the next few days with worsening respiratory failure and increased consolidation on serial chest X‐rays now also involving the right lower zone of his lungs. He was intubated, and on the same day his initial sputum sample was reported to have grown Gram‐positive branching rods resembling Nocardia on Gram stain and modified Ziehl–Neelsen (Kinyoun) stain (Fig. 2). The same organism was grown on multiple subsequent sputum samples and identified presumptively as *Nocardia cyriacigeorgica* by Bruker Biotyper Matrix‐assisted laser desorption/ionization‐time of flight (MALDI‐TOF) instrument, MA, USA. It was then referred to a separate microbiology centre and identification was confirmed by polymerase chain reaction (PCR) and DNA sequencing of the SecA1 and 16S rRNA genes. A computed tomography (CT) of the thorax was done which showed a right lower lobe necrotizing pneumonia (Fig. 2). Further CTs were done of his brain, abdomen, and pelvis which did not show any disseminated disease. He was continued on ceftriaxone 2 g daily and oral sulfamethoxazole/trimethoprim 800/160 mg twice daily, and one dose of intravenous amikacin 1 g was added to his antibiotic regimen. Ceftriaxone was subsequently changed to cilastin/imipenem 500/500 mg six‐hourly after initially failing to improve.

After four days of ventilation, he was successfully extubated and slowly weaned off supplemental oxygen and stepped down from intensive care. His steroid dose was weaned down and his antibiotic regimen has been changed to a course of per oral (PO) sulfamethoxazole/trimethoprim 800/160 mg twice daily and intravenous ceftriaxone 2 g for four weeks followed by PO sulfamethoxazole/trimethoprim for another six months as eradication therapy. He has since been discharged back to a peripheral hospital for rehabilitation.

## Discussion

Nocardia spp. are Gram‐positive aerobic actinomycetes that are found in soil and water. There have been more than a hundred different species of Nocardia identified, of which at least 33 are responsible for human diseases [3]. Pulmonary nocardiosis is an uncommon cause of pulmonary infections, with a group of five tertiary hospitals in Spain reporting only 55 cases of Nocardia pneumonia in a period of seven years [4]. In their retrospective analysis, *N. cyriacigeorgica* was the most commonly isolated species, which is the same species as what was isolated in our patient. Other species of Nocardia responsible for pulmonary diseases include *N. asteroides*, *N. brasiliensis*, *N. abscessus*, and *N. farcinia* [1, 4].

Immunosuppression and chronic lung disease are well‐established risk factors for pulmonary Nocardia infections. Immunosuppression secondary to glucocorticoid therapy, malignancy, organ transplant, primary immunodeficiency, and HIV have all been identified in a significant proportion of cases of Nocardia infections [1, 4]. Many forms of chronic lung disease also increase the risk of pulmonary nocardiosis, with Ercibengoa et al. stating that out of the 55 patients reviewed, 20 (36.4%) had chronic obstructive pulmonary disease (COPD), 16 (29.1%) had bronchiectasis, and seven (12.7%) had asthma. Nocardia infections predominantly manifest as pulmonary infections. It has been widely proposed that this is due to inhalation being the main mode of transmission of infections [1, 5]. This would explain the association between chronic lung disease and Nocardia pneumonia. Given our patient had been chronically immunosuppressed with high doses of corticosteroids and had alpha‐1 antitrypsin deficiency with severe emphysema, he had significant risk factors for Nocardia pneumonia. Interestingly, it has been reported in the literature that loss of specific host defences such as alpha‐1 antitrypsin deficiency increases the risk of infections with non‐tuberculous Mycobacterium, which are intracellular organisms [6]. As Nocardia are also intracellular pathogens, it is possible that alpha‐1 antitrypsin deficiency may have heightened his risk for nocardial infection.

As Beaman and Beaman. report, pulmonary nocardiosis can have highly variable radiological presentations, ranging from mild diffuse infiltrates, to lobar or multilobar consolidation, to necrotizing abscesses and cavitatory lesions [1]. There are no specific radiological features for distinguishing Nocardia pneumonia hence misdiagnosis may occur. Nocardia may take longer than 48 h to be grown on culture medium and more than a week to be isolated [2, 3]. Selective growth media may also be required to optimally grow Nocardia (paraffin agar, charcoal‐buffered yeast extract agar, Thayer–Martin agar and Saboraud's dextrose agar) [2, 3]. Thus, it is important to be able to recognize Nocardia as a possible cause for pulmonary infections and notify the laboratory to set up culture plates to optimally isolate the organism if suspicion is raised.

In conclusion, given the non‐specific clinical and radiological findings and difficulties in identifying Nocardia, it is important for physicians to be able to recognize and consider Nocardia as a possible cause for infections in patients with chronic lung disease who are on long‐term corticosteroids. This will lead to prompt identification and appropriate treatment of pulmonary nocardiosis.

### Disclosure Statement

Appropriate written informed consent was obtained for publication of this case report and accompanying images.

## References

[1] Beaman BL , and Beaman L . 1994 Nocardia species: host‐parasite relationships. Clin. Microbiol. Rev. 7:213–264. 8055469 10.1128/cmr.7.2.213 PMC358319

[2] Kandi V . 2015 Human Nocardia infections: a review of pulmonary nocardiosis. Cureus. 7(8):e304. 26430578 10.7759/cureus.304 PMC4571773

[3] Brown‐Elliott BA , Brown JM , Conville PS , et al. 2006 Clinical and laboratory features of the Nocardia spp. based on current molecular taxonomy. Clin. Microbiol. Rev. 19:259–282. 16614249 10.1128/CMR.19.2.259-282.2006 PMC1471991

[4] Ercibengoa M , Camara J , Tubau F , et al. 2020 A multicentre analysis of *Nocardia* pneumonia in Spain: 2010–2016. Int. J. Infect. Dis. 90:161–166. 31693939 10.1016/j.ijid.2019.10.032

[5] Steinbrink J , Leavens J , Kauffman CA , et al. 2018 Manifestations and outcomes of Nocardia infections: comparison of immunocompromised and nonimmunocompromised adult patients. Medicine (Baltimore) 97(40):e12436. 30290600 10.1097/MD.0000000000012436 PMC6200467

[6] Chan ED , Kaminska AM , Gill W , et al. 2007 Alpha‐1‐antitrypsin (AAT) anomalies are associated with lung disease due to rapidly growing mycobacteria and AAT inhibits *Mycobacterium abscessus* infection of macrophages. Scand. J. Infect. Dis. 39(8):690–696. 17654345 10.1080/00365540701225744

